# Influence of Solute Drag Effect and Interphase Precipitation of Nb on Ferrite Transformation

**DOI:** 10.3390/ma17102440

**Published:** 2024-05-18

**Authors:** Yiming Cai, Ran Wei, Duoduo Jin, Honghong Wang, Xiangliang Wan, Chengyang Hu, Kaiming Wu

**Affiliations:** 1 Collaborative Innovation Center for Advanced Steels, Wuhan University of Science and Technology, Wuhan 430081, China; circai@wust.edu.cn (Y.C.); jinduo2022@163.com (D.J.); wanxiangliang@wust.edu.cn (X.W.); huchengyang@wust.edu.cn (C.H.); wukaiming@wust.edu.cn (K.W.); 2 Department of Applied Physics, Wuhan University of Science and Technology, Wuhan 430081, China; 3 The State Key Laboratory of Refractories and Metallurgy, Wuhan University of Science and Technology, Wuhan 430081, China; wanghonghong@wust.edu.cn

**Keywords:** Nb−alloyed steels, ferrite transformation, 3D reconstruction, solute drag effect, interphase precipitation

## Abstract

The significant impact of Nb on ferrite transformation, both in terms of solute drag effect (SDE) and interphase precipitation, was investigated quantitatively. Ferrite transformation kinetics were characterized using thermal expansion experiments and theoretical calculations. The microstructures were characterized using high−temperature confocal laser scanning microscopy (CLSM), a field−emission scanning electron microscope (FESEM), and a transmission electron microscope (TEM). Under a higher driving force, interphase precipitations were observed in the sample with a higher Nb content. A three−dimensional (3D) reconstruction method was used to convert the two−dimensional (2D) image of interphase precipitation into a three−dimensional model for a more typical view. The SDE and interphase precipitation had opposite effects on the kinetics of ferrite transformation. A lower Nb content showed a strong contribution to the SDE, which delayed ferrite transformation. A higher concentration of Nb was expected to enhance the SDE, but the inhibition effect was eliminated by the interphase precipitation of NbC during interfacial migration. Both the experimental results and theoretical calculations confirmed this phenomenon.

## 1. Introduction

In recent years, with the rapid development of high−strength low−alloy (HSLA) steels, Nb has been paid more and more attention as an important micro−alloying element [1,2,3,4,5,6,7]. Nb plays an important role in influencing the ultimate microstructure and mechanical properties. The tensile strength and yield strength of Nb−micro−alloyed steel are significantly higher than those of non−Nb steel [5] and will significantly affect the microstructure and mechanical properties of the coarse−grained heat−affected zone (CGHAZ) of the weld of micro−alloyed steel, especially the impact toughness [8]. The influence of Nb on the elongation of steel is mainly caused by the interaction between Nb and C. According to the conclusion of the referenced study, in low−carbon steels, a low Nb content usually creates a high elongation, while a high Nb content produces a large amount of precipitation, leading to a low elongation [9]. The engineering application and mechanical properties of Nb−alloyed steels are inseparable from the regulation of a microstructure by phase transformation. The addition of Nb in low−carbon steels significantly affects the phase transformation kinetics of ferrite phase transformation, and two effects in general need to be emphasized: (i) Nb in a solid solution tends to segregate at the interface [1,10,11,12]; and (ii) Nb is a strong carbide−forming element [13,14,15,16,17].

The delayed effect of Nb on ferrite transformation has been the object of extensive study [2,10,12,18,19]. The large lattice mismatch between Nb and Fe is thought to be its underlying mechanism, where Nb inhibits ferrite nucleation [20,21] and growth [20] by segregation at the grain boundaries. This inhibition is referred to as the solute drag effect (SDE) of Nb [15,22,23]. The core concept of the SDE suggested that heterogeneous solute atoms with a slower diffusion segregate at the moving interface, creating a drag force, and can strongly affect the mobility of grain boundaries and, thus, the microstructure development and properties of alloys [24,25]. Among the various common substitutional alloying elements (such as Mo, Mn, Cr, Si, etc.) [11,26,27], Nb is at the top of the list. Currently, studies have quantified the SDE for Mo [27,28] and Nb [10,15] and have shown a significant impact on ferrite phase transformation. Nb precipitates can also cause delays [15]. However, compared to the delaying effect of the SDE with a pinning effect, Wang [12] showed that the SDE caused by Nb in solid solutions is much stronger than the particle−pinning effect caused by NbC.

On the contrary, the promoting effect of Nb on α−phase transformation kinetics [29,30,31] is less extensive but also reported. In previous findings, strong carbide−forming elements (such as Nb, Ti, V, etc.) precipitated to provide heterogeneous nucleation sites. These carbides reduced the local carbon concentration near austenite grain boundaries and, thus, promoted the formation of ferrite [32]. In earlier studies [33], it was suggested that the Nb in solution segregated to the austenite grain boundaries delayed the recovery and recrystallization of deformed austenite, while the defects stored in austenite provided additional intragranular nucleation sites for the ferrite phase. Fossaert [34] proposed that the possible effect of Nb in the solid solution of steel is the segregation of Nb atoms into austenite grain boundaries, which affects the nucleation of austenite transformation products. Xie [30] showed that the proportion of acicular ferrite is higher with an increase in Nb concentration. Chen [29] showed, in his conclusion, that increasing the amount of Nb(CN) precipitation promoted the formation of acicular ferrite. Similarly, in our work, we also found that a higher Nb content shows a promoting effect on ferrite transformation, but probably due to a new reason, which is the interface precipitation of NbC.

Although some general rules are accepted, in many cases, it is not easy to understand how Nb is affecting ferrite formation. The situation of Nb (precipitated or in a solution) introduces additional difficulties. 

On this basis, we provided a new understanding of the interaction of the SDE and precipitates in ferrite formation. We proposed a new research perspective that the interphase precipitation of NbC consumes the concentration of Nb at the α/γ interface and reduces the SDE. The characteristic temperatures, driving force, interfacial energy, and other kinetic parameters of niobium during interfacial migration were quantitatively evaluated. These parameters were substituted into the SDE model and the interphase precipitation model [35]. The influence of Nb on ferrite transformation was explained in comparison with experimental data. 

## 2. Experimental Procedures

**Figure 1:**
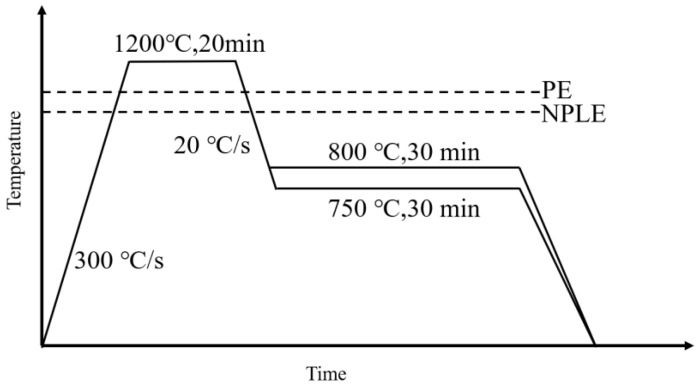
Schematic illustration of the heat treatment process.

This study investigated two Nb−containing low−carbon steels. Their composition is given in Table 1. The samples were homogenized at 1200 °C for 12 h and then shaped into lever−shaped tensile specimens with an overall length of 10 in, a working area length of 10 mm, and a diameter of 6 mm. The samples were sandpapered until the surface was smooth before they were loaded into the thermal expansion machine. Thermal expansion experiments were performed using a GLEEBLE 3500 (US) thermomechanical simulator. In the experiments, the samples were initially austenitized at 1200 °C for 20 min and rapidly cooled to 750 °C and 800 °C, holding for 30 min at each temperature. A schematic illustration of the heat treatment is shown in Figure 1. 

The heat−treated samples were vertically cut into circular slices of 1 mm thickness along the middle of the heating zone. The slices were ground, polished, and then etched with 4% nital. The morphology of the precipitated phase was observed using a field−emission scanning electron microscope (FESEM; Nova NanoSEM400, FEI, OR, US), and the composition of the precipitated phase was analyzed using energy−dispersive X−ray spectroscopy (EDS). The transmission electron microscope (TEM; JEM−F200, JEOL, JP) samples were electropolished (TenuPol−5, Struers, DK) with 10% perchloric acid and acetic acid at 20 V and 0 °C. The precipitated NbC phase was observed using a TEM.

The characteristic temperatures were calculated using the Thermo−calc software Version 2023a with the TCFE12 database. The results are presented in Table 2. The ferrite transformation kinetics were calculated using the DICTRA modules with the MoFE7 database. The average size of the prior austenite grains, obtained through high−temperature confocal laser scanning microscopy, were approximately 70 microns. The diffusion cell setup for the DICTRA simulation was half of the prior austenite grain size. DICTRA simulated the growth of the planar.

## 3. Results

### 3.1. Kinetics of Ferrite Phase Transformation

**Figure 2:**
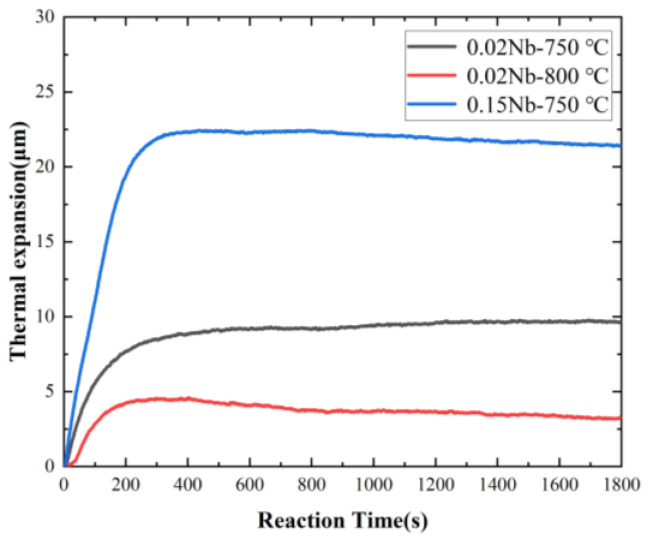
Thermal expansion curves of the alloys during the isothermal heat treatments.

**Figure 3:**
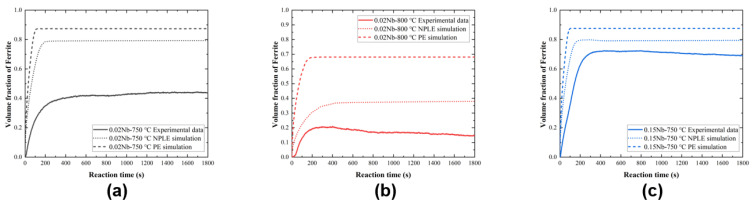
Comparison of measured and calculated volume fractions of ferrite: (**a**) 0.02 Nb−750 °C, (**b**) 0.02 Nb−800 °C, and (**c**) 0.15 Nb−750 °C. The dotted lines and the dashed lines are simulated under NPLE and PE modes by DICTRA, respectively.

The change in diameter of the alloy specimen during the isothermal process was measured as shown in Figure 2. The expansion of the specimen diameter during the isothermal process was related to the volume fraction of austenite−to−ferrite transformation, determined from the amount of expansion using the lever principle [34]. The volume fraction of ferrite with time was calculated using DICTRA under both negligible−partition local equilibrium (NPLE) and para−equilibrium (PE) models. The experimental and calculated results are compared in Figure 3.

Figure 3a,b compare the experimental results and the simulation curves of the ferrite volume fraction for the 0.02 Nb alloy at 750 °C and 800 °C, respectively. In both cases, the ferrite transformation stasis volume fraction is significantly lower than the simulated curves. However, in Figure 3c, the ferrite volume fraction for the 0.15 Nb alloy is closer to the simulation curves, and the occurrence of transformation stasis is delayed compared to the other two cases in Figure 3a,b. In previous studies [15,23,29,36], it has been observed that the Nb element, as an alloying element, is segregated to the migrating austenite/ferrite interface. This solute–interface interaction delays interface migration, leading to a phenomenon called SDE, which slows down ferrite phase transformation. When the ferrite transformation percentage stops before reaching the predicted percentage under PE conditions or NPLE conditions [23,25,37], this phenomenon is referred to as transformation stasis [38,39]. It is clear that, according to the explanation of the SDE, a higher concentration of Nb should result in more segregation and, thus, delay ferrite phase transformation. However, this is clearly inconsistent with the experimental results obtained. This phenomenon is carefully analyzed and explained in the subsequent discussion.

**Figure 4:**
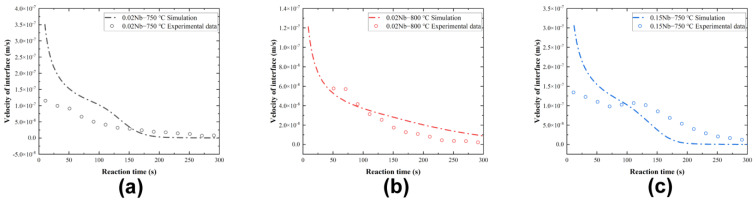
Velocity of the interface during the initial stage: (**a**) 0.02 Nb−750 °C, (**b**) 0.02 Nb−800 °C, and (**c**) 0.15 Nb−750 °C. The experimental data (dots) have been obtained after processing by the thermomechanical simulator. The dot–dash lines are simulated by DICTRA.

The comparison between the experimental and simulated velocities of the interface during the initial stage, ranging from 0 to 400 s, is shown in Figure 4. The experimental data have been obtained by processing the results in Figure 2. The experimental results are in general agreement with the simulations, except for the first few seconds. The values of the interfacial velocities are used in Section 4.1.

### 3.2. Microstructure Analysis

**Figure 5:**
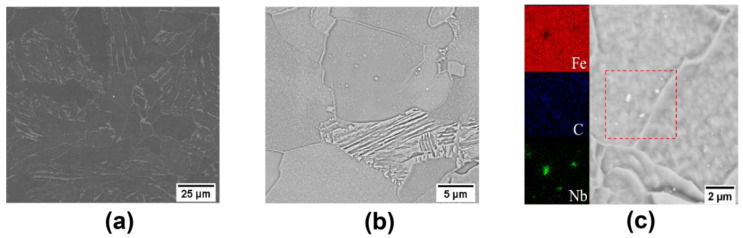
SEM images of microstructures: (**a**) 0.02 Nb−750 °C and (**b**) 0.15 Nb−750 °C, and (**c**) EDS elemental distribution in the red dashed square in the SEM image of 0.15 Nb−750 °C.

The microstructure obtained by SEM is shown in Figure 5. The SEM images of 0.02 Nb and 0.15 Nb steels clearly show the presence of precipitated phases as NbC. The density of NbC in 0.15 Nb steel is significantly higher than that in 0.02 Nb steel.

**Figure 6:**
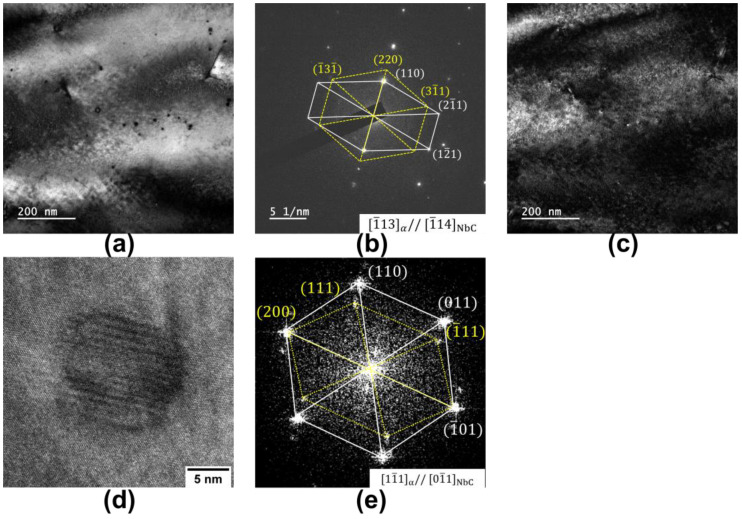
TEM characterization results of NbC precipitates in the 0.02 Nb−750 °C sample. (**a**) Bright−field image, (**b**) selected area’s electron diffraction of the precipitates in (**a**) and the (**c**) corresponding dark−field image. (**d**) High−resolution transmission electron microscopy of the NbC precipitates in (**a**,**e**) the corresponding FFT of the precipitate in (**d**).

Figure 6 presents the TEM results for the 0.02 Nb−750 °C sample containing NbC precipitates with two different orientation relationships. Figure 6a shows a bright−field image, revealing the presence of NbC precipitates. Based on the selected area’s electron diffraction pattern in Figure 6b, the corresponding dark−field image can be obtained (Figure 6c). Figure 6d,e display the images of the high−resolution transmission electron microscopy of the NbC precipitates in Figure 6a and the corresponding FFT of the precipitate.

**Figure 7:**
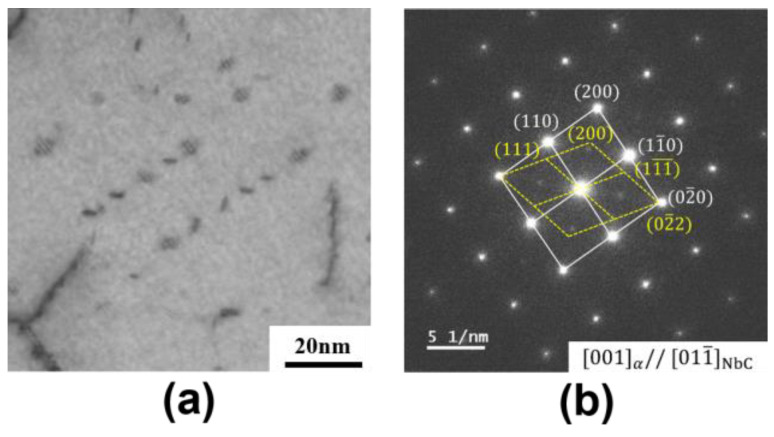
TEM characterization results of the NbC precipitates in the 0.15 Nb−750 °C sample. (**a**) Bright−field image and (**b**) the corresponding selected area’s electron diffraction pattern from (**a**).

**Figure 8:**
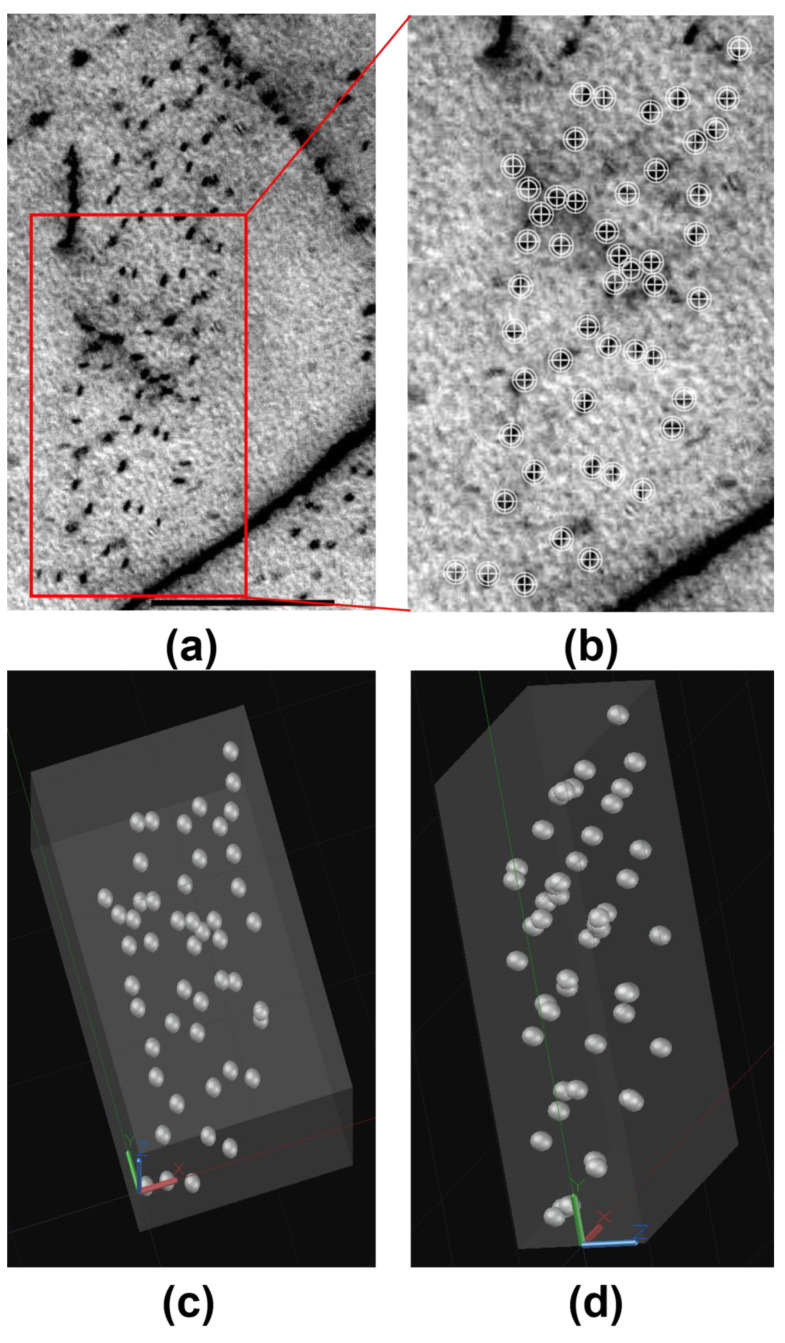
(**a**) TEM morphology of the interphase precipitation in the 0.15 Nb specimen isothermally treated at 750 °C for 30 min, and (**b**) an enlarged image of a red rectangular area in (**a**). (**c**) Three−dimensional reconstruction of the precipitation in (**b**), and (**d**) another direction’s view of the 3D reconstruction of the precipitation in (**b**).

Figure 7a displays the bright−field images of 0.15 Nb−750 °C. The distinct staircase−like interphase precipitation can be clearly observed, providing strong evidence of the interphase precipitation of NbC at high−Nb−concentration conditions. Figure 7b shows the selected area’s electron diffraction pattern, showing a variant of the B−N orientation relationship between the ferrite and the precipitation, taken from Figure 7a. According to a statistical analysis, the average length of the interphase precipitation was 6 nm, and the width was 2 nm. However, no obvious interphase precipitation features were observed in 0.02 Nb at both 750 °C and 800 °C. Figure 8 shows that a more typical image of the interphase precipitation can be obtained by means of labeling and 3D reconstruction. Figure 8a is an original STEM image, and Figure 8b is the marking of part of the NbC precipitates. By simplifying the NbC particles into spheres and rearranging them in a 3D spatial model, classical interphase precipitation can be observed. Figure 8c,d are two directions’ views of the 3D structure, respectively.

## 4. Discussion

### 4.1. The Inhibitory Effect of Nb on Ferrite Phase Transformation

#### 4.1.1. Description of the Solute Drag Effect Model

Figure 7a displays the bright−field images of 0.15 Nb−750 °C. The distinct staircase−like interphase precipitation can be clearly observed, providing strong evidence of the interphase precipitation of NbC at high−Nb−concentration conditions. Figure 7b shows the selected area’s electron diffraction pattern, showing a variant of the B−N orientation relationship between the ferrite and the precipitation, taken from Figure 7a. According to a statistical analysis, the average length of the interphase precipitation was 6 nm, and the width was 2 nm. However, no obvious interphase precipitation features were observed in 0.02 Nb at both 750 °C and 800 °C. Figure 8 shows that a more typical image of the interphase precipitation can be obtained via labeling and 3D reconstruction. Figure 8a is an original STEM image, and Figure 8b is the marking of part of the NbC precipitates. By simplifying the NbC particles into spheres and rearranging them in a 3D spatial model, classical interphase precipitation can be observed. Figure 8c,d are two directions’ views of the 3D structure, respectively.

In the past half−century, scholars have proposed [23,25,40] many classic solute drag models to quantitatively describe the SDE. Recently, Miyamoto [27] used the SDE model, which considers the co−segregation behavior of C and alloying elements, based on the energy dissipation concept of Hillert and Sundman [40], referring to the Enomoto [25,41] model to systematically study the effect of Mo and Nb on the growth of ferrite [15,27]. The calculation is carried out as follows.

**Figure 9:**
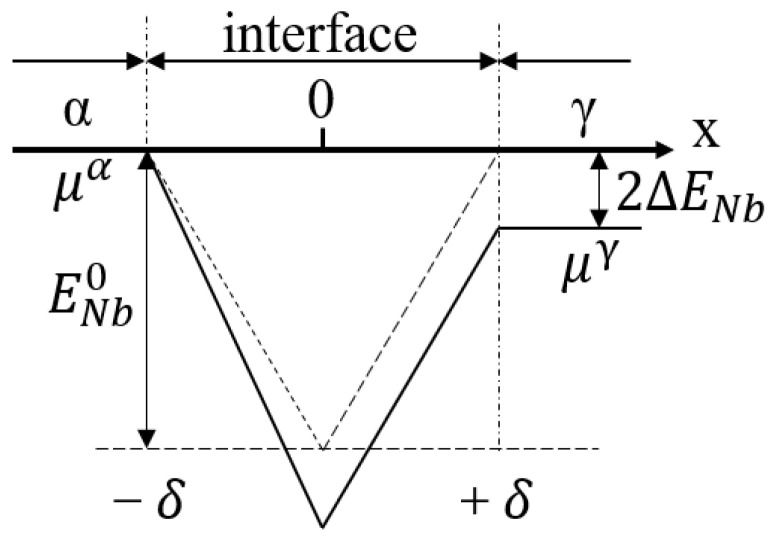
Schematic diagram of the solute chemical potential well at the α/γ interface.

The chemical potential *μ_i_* of each element can be approximated as follows [42]:

(1) $$\mu_{Fe}=RT\ln\left(\left(1-y_{Nb}\right)\left(1-y_{C}\right)\right)-y_{Nb}y_{C}W_{int}$$

(2) $$\mu_{Nb}=RTln(y_{Nb}(1-y_{C}))+(1-y_{Nb})y_{C}W_{int}+E_{Nb}(x)$$

(3) $$\mu_{C}=RTln(y_{C}/(1-y_{C}))+y_{Nb}W_{int}+E_{C}(x)$$

where $y_{i}$ is the site fraction of element *i* (*i* = C or Nb), R is a gas constant, and *T* is the temperature. $E_{i}(x)$ (*i* = C or Nb) is the binding energy between the solute atoms and the α/γ interface at the coordinate *x* (as shown in Figure 9), $E_{i}0$ is the segregation energy of the solute element, 2Δ*Ei* is the chemical potential difference of the solute atoms between the α and γ phases, 2*δ* is the interface thickness, and $W_{\mathrm{int}}$ represents the interaction coefficient of Nb−C at the interface, which is related to the Wagner coefficient ($\varepsilon_{int}$) as shown below.

(4) $$\varepsilon_{int}=\frac{W_{\mathrm{int}\;}}{RT}$$

According to reference [43], the diffusion flux of Fe, Nb, and C can be represented as follows:

(5) $$-J_{Fe}=J_{Nb}=-\frac{D_{Nb}^{\mathrm{trans}.\;}}{RTV_{m}}y_{Nb}(1-y_{Nb})\frac{d\Delta \mu }{dx}$$

(6) $$J_{C}=-\frac{D_{C}^{\mathrm{trans}.\;}}{RTV_{m}}y_{C}(1-y_{C})\frac{d\mu_{C}}{dx}$$

where $D_{Nb}^{\mathrm{trans}.\;}$ and $D_{C}^{\mathrm{trans}.\;}$ are the interfacial diffusion coefficients of Nb and C, and Δ*μ* and $V_{m}$ are the diffusion potential of Fe and Nb and the molar volume. According to Equations (1) and (2), Δ*μ* can be written as follows:

(7) $$\Delta \mu =\mu_{Nb}-\mu_{Fe}=RT\ln\left(\frac{y_{Nb}}{1-y_{Nb}}\right)+y_{C}W_{Nb-C}+E_{Nb}\left(x\right).$$

When simulating the SDE process, considering factors such as the fast diffusion rate of atoms at the interface and a thin interface thickness, it is often assumed that the solute distribution at the interface is in a steady state. At this point, the following governing equation needs to be satisfied:

(8) $$\partial (vy_{i}-J_{i}V_{m})/\partial x=0$$

where *v* represents the interface velocity. By combining the boundary conditions and substituting Equations (5)–(7) into Equation (8), we can obtain the following:

(9) $$\frac{\partial y_{Nb}}{\partial x}+\frac{y_{Nb}\left(1-y_{Nb}\right)}{RT}\frac{\partial E_{Nb}}{\partial x}+\frac{W_{\mathrm{int}\;}}{RT}y_{Nb}\left(1-y_{Nb}\right)\frac{\partial y_{C}}{\partial x}+\frac{v}{D_{Nb}^{\mathrm{trans}.\;}}\left(y_{Nb}-y_{Nb}^{\alpha }\right)=0$$

(10) $$\frac{\partial y_{C}}{\partial x}+\frac{y_{C}\left(1-y_{C}\right)}{RT}\frac{\partial E_{C}}{\partial x}+\frac{W_{\mathrm{int}\;}}{RT}y_{C}\left(1-y_{C}\right)\frac{\partial y_{Nb}}{\partial x}+\frac{v}{D_{C}^{\mathrm{trans}.\;}}\left(y_{C}-y_{C}^{\alpha }\right)=0$$

where $y_{Nb}^{\alpha }$ and $y_{C}^{\alpha }$ are the contents of Nb and C in ferrite, and $D_{Nb}^{\mathrm{trans}.}$ is assumed to be constant. Since the diffusion coefficient of C is several orders of magnitude larger than that of Nb, the last term on the left−hand side of Equation (10) can be ignored. Then, Equations (9) and (10) can be simplified into the following matrix form:

(11) $$\begin{array}{c} \left(\begin{array}{cc} 1 & \frac{W_{int}}{RT}y_{C}(1-y_{C})\\ \frac{W_{int}}{RT}y_{Nb}(1-y_{Nb}) & 1 \end{array}\right)\left(\begin{array}{c} \frac{\partial y_{C}}{\partial x}\\ \frac{\partial y_{Nb}}{\partial x} \end{array}\right)\\ =\left(\begin{array}{c} -\frac{y_{C}(1-y_{C})}{RT}\frac{\partial E_{C}}{\partial x}\\ -\frac{y_{Nb}(1-y_{Nb})}{RT}\frac{\partial E_{Nb}}{\partial x}-\frac{v}{D_{Nb}}(y_{Nb}-y_{Nb}^{\alpha }) \end{array}\right). \end{array}$$

The concentration distribution of solute atoms at a given interface velocity can be obtained. Finally, according to Equations (12) and (13) [9], the energy dissipation caused by the SDE ($\Delta G_{SDE}$, J/mol) and the segregation of Nb to the interface ($\Gamma_{Nb},{atom/nm}^{2}$) can be calculated, respectively, as follows:

(12) $$\Delta G_{SDE}=\frac{V_{m}}{v}\int_{-\delta }^{+\delta }J_{Nb}\frac{d\Delta \mu }{dx}dx$$

(13) $$\Gamma_{Nb}=\frac{1}{V_{a}}\int_{-\delta }^{+\delta }\left(y_{Nb}-y_{Nb}^{0}\right)dx.$$

In Equation (13), *V_a_* represents the effective volume of a single iron atom ($0.0118\;{nm}^{3}/atom$).

#### 4.1.2. Comparison with Experimental Data for Ferrite Growth

Table 3 summarizes the SDE model parameters used in this study. $y_{C}^{\alpha }$ is the carbon content in ferrite that satisfies the austenite PE condition, $y_{Nb}^{\alpha }$ denotes the calculated Nb content in ferrite, and $D_{\alpha }$ is the diffusion rate of ferrite. All these thermodynamic data have been obtained from the Thermo−Calc TCFE12 database. And, since the value of the interaction coefficient at the Nb−C interface is currently unknown, in this study, smaller values of $W_{\alpha }$ = −107 kJ/mol (for ferrite) and W = 0 kJ/mol have been chosen as the interaction coefficients within the NbC interface for the 0.02 Nb alloy [15]. For the 0.15 Nb alloy, the interaction coefficients chosen are 30% of the ferrite interaction coefficient by the Miyamoto method [27], $W_{\alpha }$ = −30 kJ/mol, and W = 0 kJ/mol. $E_{C}^{0}$, 2δ (4 nm), and $E_{Nb}^{0}$ are obtained from Refs. [15,25]. $D_{Nb}^{\mathrm{trans}.}$ is a fitting parameter that discussed later in this paper. The calculations are hereby carried out using the dimensionless interfacial velocity $V_{norm}=v\delta /D_{Nb}^{\mathrm{trans}.}$.

**Figure 10:**
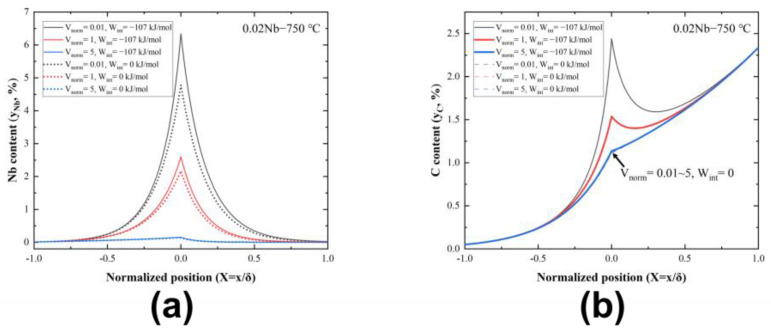
Computational results of the SDE model (using the 0.02 Nb alloy at 750 °C as an example). Concentration distribution of Nb (**a**) and C (**b**) within the α/γ interface at different interface migration rates, respectively ($V_{norm}$ is the dimensionless interface velocity, and $W_{int}$ represents the interaction coefficient of Nb−C at the interface).

Taking the example of a 0.02 Nb alloy’s transformation at 750 °C, Figure 10 illustrates the concentration profiles of Nb and C on the α/γ interface at different values of dimensionless interface velocity ($V_{norm}$). The solid and dashed lines represent the cases where the energy parameter (W) is −107 kJ/mol and 0 kJ/mol. Figure 10a shows that Nb accumulation increases at a slower migration rate. Noticeably, the stronger Nb−C interaction produces a larger segregation. 

**Figure 11:**
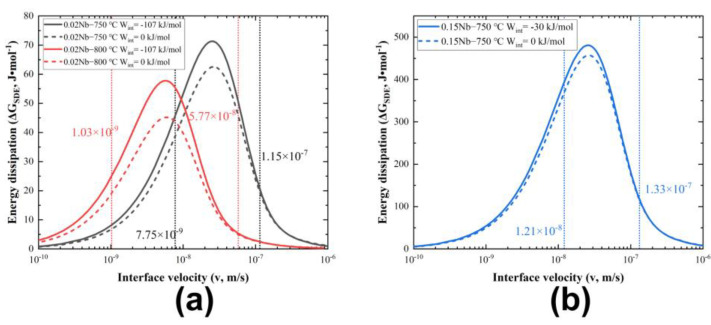
Relation between the energy dissipation due to SDE ($\Delta G_{SDE}$) and *v*, compared with the experimental diffusion rates, (**a**) 0.02 Nb−750 °C/800 °C, and (**b**) 0.15 Nb−750 °C. The dotted lines in the figures show the ranges of experimental interface velocity (interphase precipitation is not considered in the calculation).

**Figure 12:**
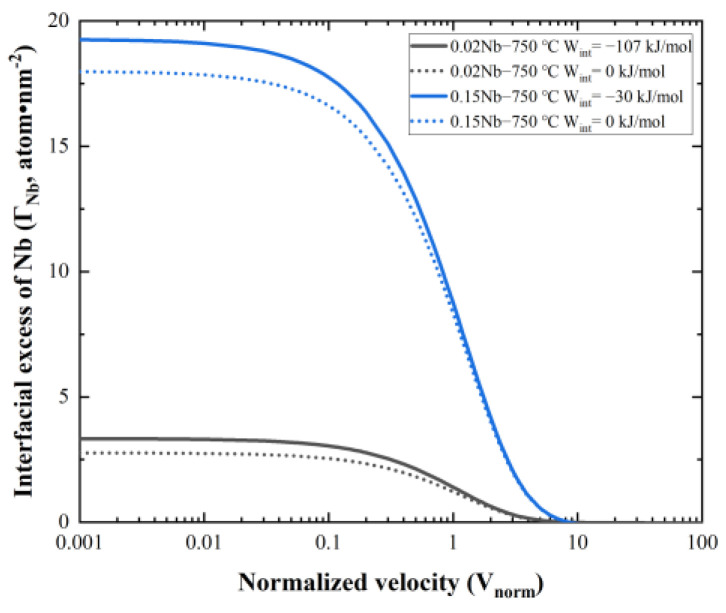
Interfacial excess of 0.02 Nb and 0.15 Nb as a function of normalized velocity (*V_norm_*) at 750 °C (interphase precipitation is not considered in the calculation).

Figure 11 analyzes the relation between the energy dissipation due to SDE ($G_{SDE}$) and *v*, compared with experimental data from different groups. The dotted lines in the figures show the ranges of the experimental interface velocity. Unfortunately, there are no reported experimental interfacial diffusion coefficients ($D_{Nb}^{\mathrm{trans}.}$). Suehiro et al. [44] have attempted to use 10 times the bulk diffusion coefficient of Nb in ferrite as $D_{Nb}^{\mathrm{trans}.}$. In this study, the simulation results have been calculated using $D_{Nb}^{\mathrm{trans}.}$~$D_{\alpha }$, where $D_{\alpha }$ is the calculated bulk diffusion coefficient of Nb and matches well with the experimental data at 800 °C in this work. However, when the temperature is lowered to 750 °C, a value of $D_{Nb}^{\mathrm{trans}.}$~20$D_{\alpha }$ is needed to obtain a better fit for the experimental results. Figure 12 provides a comparison of the interfacial excess of Nb of 0.02 Nb and 0.15 Nb, both at 750 °C. It is evident that the interfacial excess of Nb in 0.15 Nb is significantly higher than that in 0.02 Nb. The segregation level at the interface plays a crucial role in the SDE, according to previous studies [15]. Such a high segregation in 0.15 Nb is a necessary condition for interphase precipitation formation. It is a competing process between the solute segregation at the interface and the formation of interphase precipitates resulting in the temporary reduction in solute at the migrating interface. In the calculations, interphase precipitation is not considered. The segregation level at the interface is affected by the precipitation of NbC. This is discussed in Section 4.2.

Based on the above analysis, it was found that the energy dissipation was less affected by temperature when the concentration of Nb solute was small. In contrast, at the same temperature, the energy dissipation was significantly increased by the concentration. The energy dissipation value for 0.15 Nb was much larger than that for 0.02 Nb at a similar interfacial velocity, which should have shown a more pronounced effect of delaying ferrite transformation. However, this contradicts the experimental results shown in Figure 3. A rational explanation for this contradiction is presented in Section 4.2 on interphase precipitation.

#### 4.1.3. Energy Dissipation of Pinning Effect

In order to verify the energy dissipation relation between the solute drag effect and the pinning effect mentioned by Wang, the interaction between the precipitated phase and the moving α/γ interface was analyzed using the classical Zener model, and the pinning force of NbC was estimated.

(14) $$\Delta G_{PF}=3f\cdot \sigma \cdot V_{m}/2r$$

where f is the NbC volume fraction, calculated by Thermo−Calc, σ is the interface energy of the incoherent α/γ interface ($0.8\;J/m^{2}$ is used here), $V_{m}$ is the molar volume of Fe (7.0 × 10^−6^ $m^{3}$/mol), and r is the radius of NbC.

Taking the 0.15 Nb alloy as an example, the simulated volume fraction of NbC (*f*) was $2.3\times 10^{-4}$, and the equivalent radius (*r*) measured in the experiment was 2 nm ($r=\sqrt[{3}]{l_{1}^{2}\cdot l_{2}}$, where $l_{1}$ and $l_{2}$ are the major and minor axes of the NbC particles). Consequently, the calculated value of the pinning force ($\Delta G_{PF}$) was 0.97 J/mol. Therefore, the pinning effect produced by Nb was not the main cause of the slow growth of ferrite.

### 4.2. The Impact of NbC Interphase Precipitation on Ferrite Phase Transformation

In a large number of previous studies [31,33,36], it was found that there have been reports about the promotion of ferrite phase transformation by the related Nb element. The main reason is that Nb segregation to austenite grain boundaries in a solution delays the recovery and recrystallization of deformed austenite [1], while the defects stored in austenite provide additional intragranular nucleation sites for the ferrite phase and promote the formation of ferrite. Interphase precipitation [45] mostly occurs during the transformation of austenite (γ) to ferrite (α) when alloying carbides nucleate periodically in patches at the migrating α/γ interface [46]. Sakuma and Honeycombe [47] studied the effect of transformation temperature using Nb−added mild steel and found that NbC does not undergo interphase precipitation at higher or lower temperatures. This indicated that an excess or insufficient driving force for precipitation can suppress interphase precipitation. In conjunction with the above (Figure 7), it has been demonstrated that the interphase precipitation of NbC occurs in 0.15 Nb, while the phenomenon does not occur in 0.02 Nb. It is reasonable to speculate that Nb affects ferrite phase transformation due to the formation of the interphase precipitation of NbC. In this regard, this study calculated the interphase precipitation nucleation rate and the precipitation number density of NbC from the perspective of Zhang’s [35] theoretical model.

**Figure 13:**
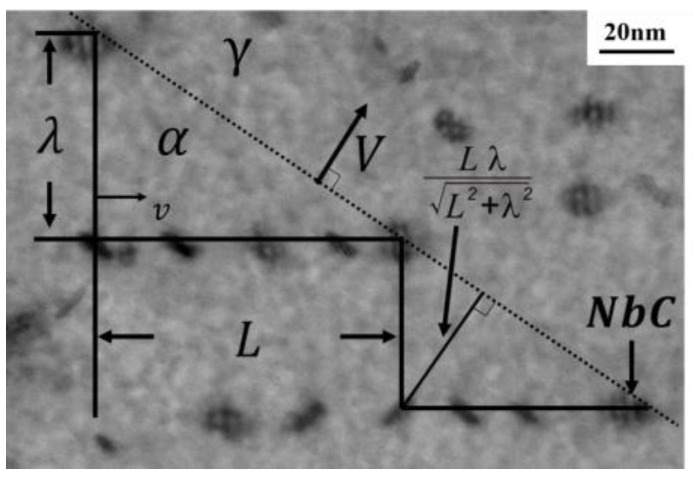
Schematic of NbC interphase precipitation with ledge−wise α growth.

The growth rate determines the duration of aging, which refers to the time when precipitation occurs at the α/γ interface during migration. As depicted in Figure 13, v represents the transverse velocity at each edge, while V denotes the apparent macroscopic growth rate. The aging time of the interface is estimated to be required for the upper ledge to cover the lower ledge. The upper ledge moves at the distance of the terrace length, *L*. The macroscopic interface distance moves forward proportionally to the ledge height (inter−sheet spacing), λ. For interphase precipitation occurring at the migrating α/γ interfaces, $t_{\mathrm{int}\;}$ is as follows:

(15) $$t_{\mathrm{int}\;}=\frac{L}{v}=\frac{\frac{L\lambda }{\sqrt{L^{2}+\lambda^{2}}}}{V}.$$

In our study, the average ratio of ledge length (L~66 nm) to height (λ~44 nm) was calculated using what had been measured experimentally. In order to elucidate the effect of the growth rate, the heterogeneous nucleation of the NbC phase at the α/γ interface was considered. Supersaturation should decrease continuously with time through the consumption of Nb and C during nucleation. The functional formula of the driving force with time was thus obtained.

(16) $$\Delta G_{m}^{NbC\left(t\right)}=\mu^{Nb\left(t\right)}+\mu^{C\left(t\right)}-G^{NbC}$$

where $\Delta G_{m}^{NbC\left(t\right)}$ represents the molar driving force for NbC precipitation, $\mu^{Nb\left(t\right)}$ and $\mu^{C\left(t\right)}$ are the chemical potentials of Nb and C in the solution before precipitation, and $G^{NbC}$ is the free energy of the NbC phase. The driving force for NbC precipitation under the NPLE model calculated in the Thermo−Calc software Version 2023a was used.

The instantaneous nucleation rate (m^−3^s^−1^) and critical radius (m) can also be rewritten as a time function:

(17) $$I^{\left(t\right)}=N^{\left(t\right)}\beta^{\left(t\right)}Z^{\left(t\right)}exp\left(-\frac{\Delta G^{*}\left(t\right)}{kT}\right)$$

(18) $$r^{*\left(t\right)}=\frac{\left(2\sigma_{\frac{\alpha }{NbC}}+2\sigma_{\frac{\gamma }{NbC}}-\sigma_{\frac{\alpha }{\gamma }}\right)V_{m}^{NbC}}{2\Delta G_{m}^{NbC}\left(t\right)}$$

where k is the Boltzmann constant (J/K), *T* is the temperature (K), σ is the interfacial energy (J/m^2^), $V_{m}^{NbC}$ is the molar volume (1.35 × 10^−5^ m^3^ /mol for NbC), while $N^{\left(t\right)}$ is the density of the nucleation site ($m^{-3}$) taken to be the density of the Nb atoms, $\beta^{\left(t\right)}$ is the frequency factor ($s^{-1}$), $Z^{\left(t\right)}$ is the Zeldovich factor (dimensionless), and $\Delta G^{*}\left(t\right)$ is the activation energy for nucleation (J), given by

(19) $$N^{\left(t\right)}=\frac{X_{Nb}^{\left(t\right)}}{V_{m}^{\alpha }/N_{A}}$$

(20) $$\beta^{*\left(t\right)}≅\frac{4\pi {\left(r^{\left(t\right)}\right)}^{2}X_{Nb}^{\left(t\right)}D_{Nb}}{a^{4}}$$

(21) $$Z^{\left(t\right)}=\frac{V_{m}^{NbC}/N_{A}{\left(\frac{\Delta G_{m}^{NbC}\left(t\right)}{V_{m}^{NbC}}\right)}^{2}}{8\pi {\left(kT\sigma_{\frac{\alpha }{NbC^{3}}}\right)}^{\frac{1}{2}}}$$

(22) $$\Delta G^{*}\left(t\right)=\frac{\pi {\left(2\sigma_{\frac{\alpha }{NbC}}+2\sigma_{\frac{\gamma }{NbC}}-\sigma_{\frac{\alpha }{\gamma }}\right)}^{3}}{12}{\left(\frac{V_{m}^{NbC}}{\Delta G_{m}^{NbC}\left(t\right)}\right)}^{2}$$

where $X_{Nb}^{\left(t\right)}$ is the atomic fraction of Nb, the distance between the nearest substituted atoms in α is (a = 0.248 nm), and $N_{A}$ is Avogadro’s constant (${mol}^{-1}$). As discussed above, the number density of the NbC formed by interphase precipitation can be simply estimated as a time−dependent nucleation rate integrated over time,

(23) $$\rho =\int_{0}^{t}I^{\left(t\right)}dt.$$

In the simulations of the above equations, it was clearly observed that the nucleation rate and number density of the NbC phase separation process were strongly affected by the interface energy used. In order to simplify the calculation, 0.8 J/m^2^ was used for the interfacial energy of the α/γ interface [48]. According to the theoretical study conducted by Yang and Enomoto [49,50], when there is a cube–cube orientation relationship between NbC and austenite, the interface energy of the (001) crystal face is about 3 J/m^2^, while the interfacial energy between NbC and ferrite with a B−N orientation relationship is approximately 1.8 J/m^2^. 

**Figure 14:**
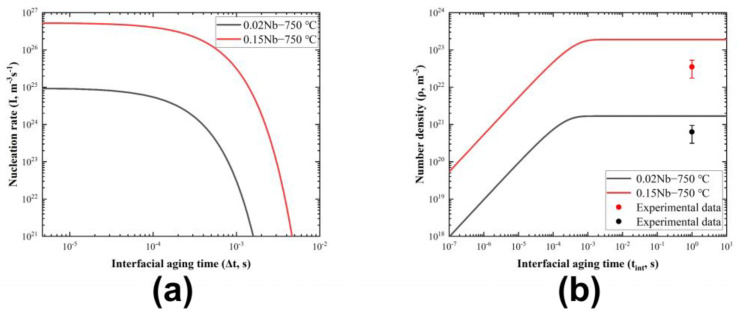
Variations in (**a**) nucleation rate and (**b**) NbC number density with interface aging time in isothermal phase transformation of 0.02 Nb and 0.15 Nb alloys at 750 °C. The experimental data are the number density estimated from TEM images with an observation area thickness of 50–100 nm.

In Figure 14, variations in the nucleation rate and NbC number density with interface aging time of the 0.02 Nb and 0.15 Nb alloys at 750 °C are shown, based on the calculated interfacial energies described above. Clearly, the nucleation rate is greatly affected by the driving force for precipitation. At the same phase transformation temperature, the Nb concentration is the dominant factor in determining the driving force. The nucleation rate of 0.15 Nb is several orders of magnitude higher than that of 0.02 Nb. On the other hand, the presence of interphase precipitation reduces the degree of supersaturation, leading to a continuous decrease in the nucleation rate with increasing aging time. By integrating the nucleation rate, Figure 14b shows the calculated number density of NbC interphase precipitation. The experimental data in the figure are obtained by counting the amount of precipitates in six STEM images of each component sample to obtain the average surface density. Combined with an observation area thickness of the TEM sample of 50–100 nm, the number density was estimated.

In order to calculate the segregation of Nb during interphase precipitation, the volume of the specified interface region was assumed to be V (with an interface width of 4 nm, denoted as 2δ, and the interface cross−sectional area as *S*, V=2δ·S). The total number of NbC particles (*n*) in the region was determined as

(24) n=ρ·V.

Concurrently, based on the NbC morphology in the image, we postulated the individual volume of a single NbC particle as $V_{NbC}^{*}$:

(25) $$V_{NbC}^{*}=4\pi abc/3$$

assuming an ellipsoid with *a* = 3 nm and *b* and *c* = 1 nm.

(26) $$n_{mol}=n\cdot \frac{V_{NbC}^{*}}{V_{m}^{NbC}}$$

where $n_{mol}$ is the total number of moles of NbC in the region and $V_{m}^{NbC}$ is the molar volume. The interfacial excess of Nb during interphase precipitation $\Gamma_{Nb}$ is obtained as follows:

(27) $$\Gamma_{Nb}=n_{mol}\cdot \frac{N_{A}}{S}=2\delta \cdot \rho \cdot \frac{V_{NbC}^{*}}{V_{m}^{NbC}}\cdot N_{A}.$$

The $\Gamma_{Nb}$ during the interphase precipitation of 0.15 Nb was calculated to be 3.81 × 10^35^, which was much greater than the segregation amount of Nb during the SDE.

Therefore, it can be concluded that the interphase precipitation of NbC occurs in the 0.15 Nb alloy at the migrating α/γ interface, effectively weakening the SDE influence on ferrite growth. However, no interphase precipitation occurs in the 0.02 Nb alloy, so ferrite phase transformation is suppressed by the SDE. This conclusion is consistent with the results presented in Figure 3, thus providing strong evidence that the interphase precipitation of NbC significantly affects the ferrite phase transformation process.

## 5. Conclusions

This study quantitatively investigated the effect of Nb on isothermal ferrite transformation. It aimed to elucidate the role of Nb in the phase transformation of ferrite, both in terms of interphase segregation and interphase precipitation.

1. In our study, 0.15 wt.% Nb steel was closer to the NPLE and PE models than 0.02 wt.% Nb steel during ferrite phase transformation. The sample of 0.02 wt.% Nb steel showed a strong effect of solute drag on ferrite growth, which delayed ferrite transformation.
2. Interphase precipitation was observed in 0.15 wt.% Nb, while only random precipitation was observed in 0.02 wt.% Nb.
3. The 0.15 wt.% Nb steel sample was expected to enhance the SDE, but the inhibition effect was eliminated by the interphase precipitation of NbC during interfacial migration. Both the experimental results and the theoretical calculations confirmed this phenomenon.
4. The conclusions from the SDE model and the interphase precipitation model suggest that the growth kinetics of ferrite can be significantly suppressed by the addition of a small amount of Nb element. However, the interphase precipitation of NbC occurs in the presence of a higher concentration of Nb, which depletes the Nb content at the α/γ interface and eliminates the inhibitory effect of solute drag on ferrite growth.

Due to the complex characteristics of Nb segregation and precipitation in steel, the synergistic and competitive relationship between its segregation and precipitation needs to be further systematically investigated.

## Figures and Tables

**Table 1:** Chemical compositions (wt.%) of the alloys used in this study.

Alloys	Fe	Nb	C	Mn	Si	P	S
0.02 Nb	Bal	0.02	0.062	1.0	0.21	0.008	0.002
0.15 Nb	Bal	0.15	0.062	1.0	0.21	0.008	0.002

**Table 2:** Corresponding characteristic temperatures.

Alloys	Ae_3_ (°C)	PE (°C)	NPLE (°C)	Solution Temp. of NbC in ɣ (°C)
0.02 Nb	860	834	818	1023
0.15 Nb	866	838	824	1207

**Table 3:** Parameters used in the present SDE model.

Variables	0.02 Nb−750 °C	0.02 Nb−800 °C	0.15 Nb−750 °C
$\Delta E_{C}/kJ/mol$	−16.4 kJ/mol	−15.6 kJ/mol	−17.7 kJ/mol
$\Delta E_{Nb}/kJ/mol$	−1.4 kJ/mol	−0.1 kJ/mol	−1.4 kJ/mol
$W_{Nb-C}/kJ/mol$	−107 kJ/mol		
$E_{C}^{0}/kJ/mol$	10 kJ/mol		
$E_{Nb}^{0}/kJ/mol$	50 kJ/mol		
2δ/nm	4 nm		
$D_{\alpha }$	$2.04\times 10^{-18}$	$8.78\times 10^{-18}$	$2.04\times 10^{-18}$
$D_{Nb}^{\mathrm{trans}.}$	$20D_{\alpha }$	$D_{\alpha }$	$20D_{\alpha }$
$y_{C}^{\alpha }$	$5.06\times 10^{-4}$	$2.81\times 10^{-4}$	$5.46\times 10^{-4}$
$y_{Nb}^{\alpha }$	$1.2\times 10^{-4}$	$1.2\times 10^{-4}$	$9.0\times 10^{-4}$

## Data Availability

Data are contained within the article.
